# Acute Fulminant Pancreatitis: Debridement or Formal Resection of the Pancreas

**DOI:** 10.1155/1993/97832

**Published:** 1993

**Authors:** Heikki Kiviniemi, Jyrki MäKelä, Matti I. Kairaluoma

**Affiliations:** Department of Surgery Oulu University Central Hospital Kajaanintie 50 Oulu SF-90220 Finland

## Abstract

During the ten year period from 1980 to 1989, 51 patients were treated at Oulu University Central Hospital for fulminant acute pancreatitis. Five were in a moribund state on admission and died shortly afterwards, 6 were treated conservatively and survived, and 40 were operated on, 17 by primary pancreatic resection and 23 by debridement of the peripancreatic area. Mortality rates were 53 per cent for the resection group and for the debridement group 22 per cent.

Reoperations were performed in 24 per cent of patients in the pancreatic resection group and in 60 per cent of those in the debridement group.

The high mortality rate associated with primary pancreatic resection has caused us to adopt a more conservative strategy, and surgical treatment is directed towards later complications of this severe disease.


*HPB Surgery*, 1993, Vol. 6, pp. 245–254
Reprints available directly from the publisher
Photocopying permitted by license only

© 1993 Harwood Academic Publishers GmbH
Printed in the United States of America

### REVIEW

### BILIARY SURGERY VIA MINILAPAROTOMY — A LIMITED PROCEDURE FOR BILIARY LITHIASIS

TAKUKAZU NAGAKAWA
*The Second Department of Surgery, School of Medicine, Kanazawa University, Kanazawa, Japan*

*(Received 22 September 1992)*

Cholelithiasis until now has been treated using solvents, lithotripsy via a biliary endoscope, laser or shock wave lithotripsy, and laparoscopic cholecystectomy. I have developed a new surgical treatment for cholelithiasis in which a cholecystectomy is performed through a minilaparotomy. This paper presents this new technique and discusses the principles of surgery for cholelithiasis using this technique. This procedure is performed by a 2 to 3 cm subcostal skin incision in the right hypochondrium. More than 400 patients were treated by this technique. This procedure is not different in terms of blood loss or operation time from conventional methods, and no significant complications have occurred. Intraoperative X-ray examination is performed routinely because of easy insertion of a tube from the cystic duct into the bile duct. Reduction of the length of the incision greatly facilitates postoperative recovery, shortening the hospital length-of-stay to within 3 days. The surgical manipulation of only a limited area of the upper abdomen is unlikely to induce postoperative syndromes, such as adhesions or ileus. Following this experience, a biliary drainage procedure based on cholangionmanomery and primary closure of the choledochotomy was introduced. This approach allowed even patients with choledocholithiasis to undergo a minilaparotomy and be discharged within one week.

KEY WORDS: Cholecystectomy, minilaparotomy, variable loading cholangiomanometry, biliary drainage procedure, primary closure of the choledochotomy

### INTRODUCTION

Cholelithiasis is one of the most common digestive diseases. Until recently, no alternative to laparotomy was available, however cholelithiasis can be treated using solvents[1]. Lithotripsy via biliary endoscopy[2], laser or shock wave lithotripsy[3], and laparoscopic cholecystectomy[4]. These advances have limited the indications for the classical surgical approach to the treatment of gallstones to relatively few patients. Accordingly, I have developed a new surgical treatment for cholelithiasis in which a cholecystectomy is performed through a minilaparotomy. This paper presents this new technique and discusses the principles of surgery for cholelithiasis using this technique.

Address correspondence to: Takukazu Nagakawa, M.D., The Second Department of Surgery, School of Medicine, Kanazawa University, 13-1 Takara-machi, Kanazawa 920, Japan

### *Surgery for Cholelithiasis through a One-Inch Minilaparotomy*

### *Preoperative preparation*

The success of using a limited incision for biliary surgery depends upon having a reliable description of the entire biliary system preoperatively. Gallstones need to be localized, and their number and characteristics determined by abdominal ultrasonography. Cholangiograph is essential to define the anatomy of the entire biliary system. If endoscopic retrograde cholangiopancreatography (ERCP) does not visualize the gallbladder well, we routinely perform selective percutaneous transhepatic cholecystography (s-PTC). Since our procedure does not permit close intraoperative examination of the peritoneal cavity, an upper gastrointestinal series and a test for occult blood test are essential.

### *Operative technique*

A 2- to 3-cm skin incision is made subcostally in the right hypochondrium, over the cystic duct of the gallbladder (Figure 1). The anterior sheath of the rectus abdominal muscle is incised parallel to the skin incision. The peritoneum is exposed by muscle splitting, not transecting, the rectus abdominal muscle, and the peritoneum is opened longitudinally. The cystic duct is grasped and occluded temporarily with either small forceps, which we have designed, or a hemoclip to prevent stones from migrating into the common bile duct during surgical manipulation and to

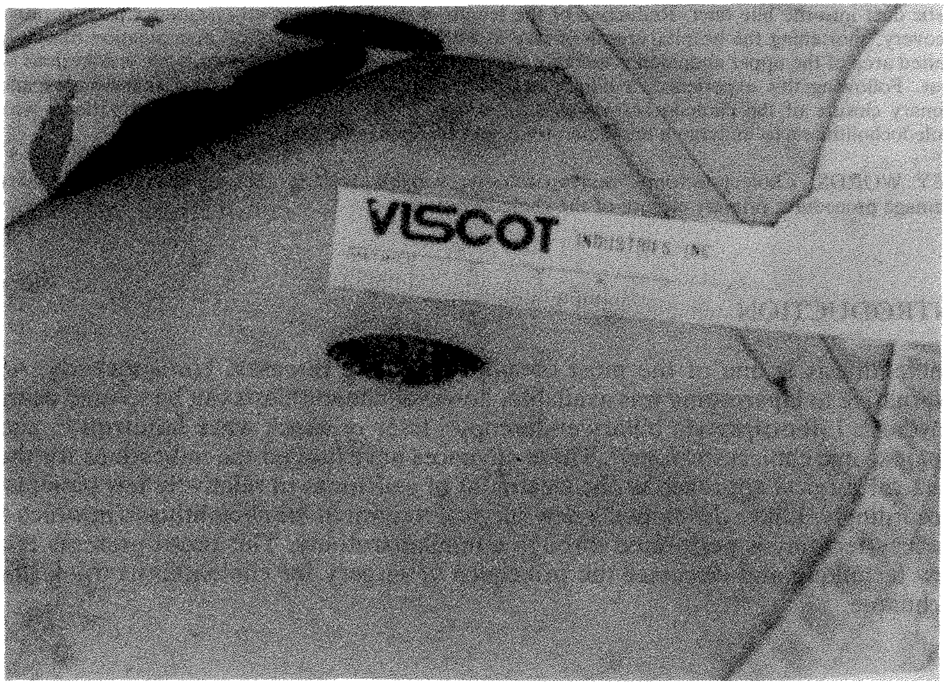

**Figure 1** One-inch skin incision for cholecystectomy.

prevent bleeding from the gallbladder wall (Figure 2). Detachment of the gallbladder from the gallbladder bed is facilitated by exerting traction on the gallbladder bed to make it more shallow. The visual field is illuminated with the light of a laparoscope from outside. The detachment is easier when the contents of the gallbladder have been aspirated. Bleeding from the gallbladder bed is controlled using electric coagulation. The cystic artery is grasped and oculuded by a hemoclip. The cholangiogram should be available in the operating room to answer any questions about the anatomy of the cyatic duct.

Once the gallbladder has been detached completely, cholangiomanometry and chlolangiography are performed through a tube inserted into the cystic duct. Both these procedures must be performed in this technique to be certain that bile flow is unimpeded and there are no residual stones. Patients with choledocholithiasis undergo choledochotomy and lithotomy. Cholangiomanometry and cholangiography are performed using an M-N tube which was designed to enable saline or contrast medium to be introduced into the bile duct without suture of the opened bile duct. Patients with abnormal biliary pressures require a biliary drainage procedure, as discussed later. Three intraoperative cholangiograms are taken with the patient in different positions, and using different concentration, of contrast medium to detect residual stones.

Initially, we routinely inserted T-tubes in all patients who had undergone choledochotomy and lithotomy. However, we now perform primary closure without intubation when the biliary pressure is normal and the likelihood of residual

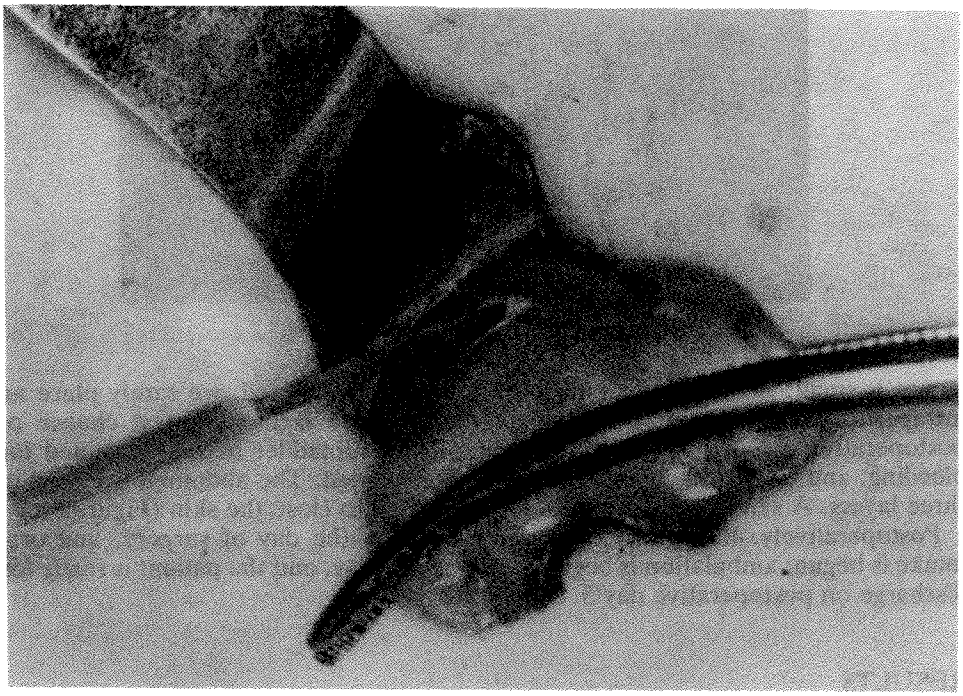

**Figure 2** The gallbladder is picked up and separated from the liver by electrocautery. Once the gallbladder is free, the cystic artery is clamped by a hemoclip.

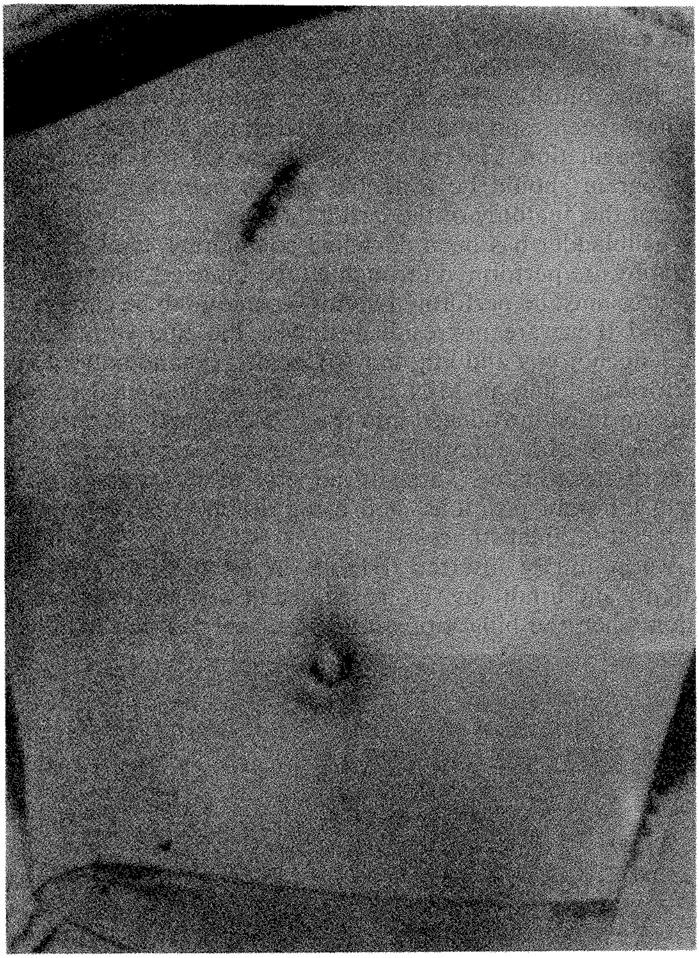

**Figure 3** Skin closure.

stones is small. If only cholecystectomy has been performed, we rarely place an intraperitoneal drain, but we do place one if there is the slightest chance of postoperative hemorrhage or bile leakage. The gallbladder bed is examined for bleeding, and electrocoagulation performed as needed. The abdomen is closed in three layers. A skin stapler and skin tape are used to close the skin (Figure 3).

Postoperatively, the gastric tube is removed on the day of surgery, and oral intake is begun; ambulation is begun the following day, and the patient is ready for discharge on postoperative day 3.

### RESULTS

We have treated more than 400 patients with cholelithiasis by minilaparotomy (Table 1). The length of incision has varied slightly, depending on the operator's

**Table 1** Comparison of standard cholecystectomy and chotecystectomy by minilaparotomy

| | *Minilaparotomy (N= 103)* | *Standard cholecystectomy (N= 106)* |
|---|---|---|
| Blood loss | 103±103 mL | 86±76 mL |
| Time of operation | 112±28 min | 96±28 min |
| Length of hospitalization | 9.7±3.4 days | 15.6±4.1 days |

experience, but the present procedure is not different in terms of blood loss or time of operation compared with conventional methods, and no significant complications have occurred. The present method has definitely shortened the length of hospitalization. However, less than 20 have been discharged within 3 days because of patient and attending physician resistance, as well as social reasons related to insurance in Japan.

### *Advantages of the Present Procedure*

Reduction of the length of the incision facilitates postoperative recovery greatly, shortening the hospital length-of-stay. This, in turn, minimizes the patient's financial burden. Financially, surgical manipulation of only a limited area of the upper abdomen is less likely to induce postoperative syndromes, such as adhesion or ileus.

### *Cautions for the Present Procedure*

Intraoperative cholangiography is essential, as mentioned earlier. Precise localization of gallstones is necessary to select the surgical procedure and reduce the risk of retained stones. Retained biliary stones have posed a serious problem in the past[5]. Intraoperative cholangiography, which is now routine in most hospitals in Japan, and postoperative interventional radiography have decreased the morbidity and mortality of this procedure greatly. Still, the conditions under which intraoperative cholangiography is performed are less than ideal. We take three cholangiograms to reduce the risk of missing a retained stone (Figure 4), but it is risky to depend entirely on intraoperative cholangiograms for information about biliary anatomy. Therefore, preoperative imaging, using a variety of projections, including the prone position, is essential.

Injuries to the biliary tract which result from surgery for cholelithiasis occur rarely, but the morbidity they cause is significant. Most injuries can be prevented by defining the anatomy of the entire biliary system, including the confluence of the cystic duct preoperatively. The limited exposure which a minilaparotomy affords highlights the importance of defining the anatomy beforehand.

Preoperative examination is also necessary to exclude the existence of concomitant lesions, especially malignant tumors of the biliary tract, which require a completely different treatment. Finally, cholangiography can identify abnormal junctions between the pancreatic and common bile ducts, as well as less common lesions, such as, para-papillary diverticulum, which can promote the formation of gallstones.

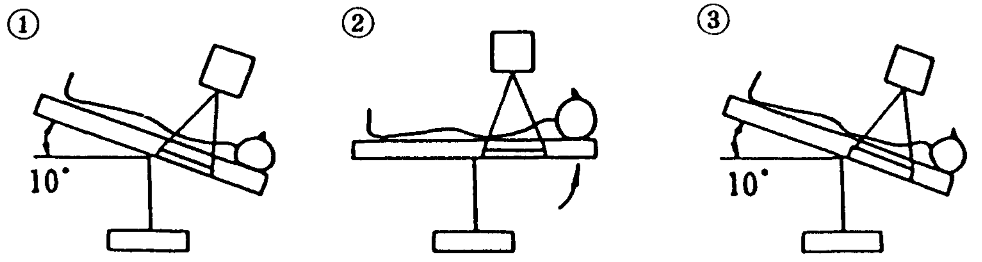

**Figure 4** Operative cholangiography uses a series of three films. 1. With the patient in a 10 degrees head-down position, 20 to 40 mL of 30% Urographin is injected. 2. Radiograph is exposed with the patient supine, without contrast agents, and 3. With the patient in a 10 degrees head-down position, 10 mL of 60% Urographin is injected.

### *Surgical Indications for Asymptomatic Cholelithiasis*

We usually operate on patients with cholecystolithiasis immediately after the diagnosis is made, regardless of whether or not they are symptomatic. When the patient's general medical condition is so poor that surgery seems dangerous, our initial treatment is conservative. It is our experience that most patients with asymptomatic cholelithiasis eventually become symptomatic, and the risk of cancer of the gallbladder is not trivial.

Furthermore, most patients with gallstones have functional abnormalities of the gallbladder, such as bile retention and concentration secondary to loss of epithelium as a result of chronic cholecystitis.

On the other hand, many patients in other hospitals are managed medically, by administering solvents. In addition, other noninvasive therapies for gallstones have been developed recently and are used to treat patients with asymptomatic cholelithiasis. However, these options have limitations. Since most such treatments preserve the gallbladder, gallstones recur, and chronic cholecystitis and cancer of the gallbladder are likely to occur. Cholecystectomy remains the definitive treatment of choice for asymptomatic cholelithiasis, especially since a minilaparotomy greatly reduces the burden to the patient.

### *Indications for Biliary Drainage Procedure*

### *Historical background*

Recurrence of gallstones following lithotomy for choledocholithiasis was recognized early in the history of biliary surgery. Various methods of prophylaxis have been tried. Procedures involving the lower biliary tract, especially duodenal sphincteroplasty, have been employed to reduce bile stagnation, presumed to be due to obstruction at the papilla. Bile stagnation has been shown to induce epigastric pain and cholangitis, and failure of the papilla to prevent reflux also has been reported to cause symptoms. However, advances in cholangiomanometry and knowledge of the dynamics of the papillary region have led to greater understanding of the papilla's function. This in turn has resulted in a re-evaluation of the role of biliary drainage[6].

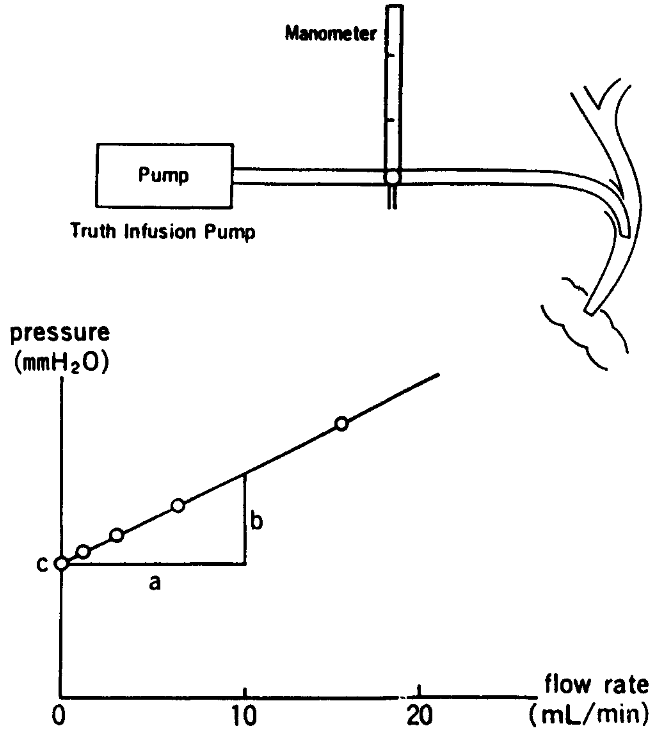

**Figure 5** Measurement of biliary pressure by the variable loading method. Resistance (R) = b/a unit (mm $H_2O$/mL/min.) — intraluminal resistance, residual pressure at zero flow rate (P) = mm $H_2O$. Normal: R = 1 to 7 units, P = 50 to 150 mm $H_2O$.

*Indications for a biliary drainage procedure based on cholangiomanometry*

The indications for biliary drainage procedure vary between hospitals. "Poor papillary passage" has been semi-quantified based on the passage of a biliary bougie of standard caliber and drainage of contrast material into the duodenum during intraoperative cholangiography. The presence of a large number of stones in the common bile duct, advanced cholangiectasis and pigmented gallstones also have been suggested as indications for a biliary drainage procedure. However, these criteria are less objective and may lead to additional, unnecessary procedures.

We perform variable loading cholangiomanometry intraoperatively to determine whether a drainage procedure is needed. This procedure has been outlined in detail elsewhere. Briefly, physiologic saline is injected in 5 steps of increasing rate through a tube introduced through the cystic duct, a T-tube, or an M-N tube, and the perfusion pressure is measured at each rate (Figure 5). The resistance (R) and residual pressure (P) are calculated.

On the basis of the R and P values and the responses to the administration of various drugs, we have divided papillary dysfunction into 4 types: organic papillary stenosis, functional papillary stenosis, papillary hypofunction, and other causes of papillary dysfunctions, such as para-papillary diverticulum, anomalous union of the bile duct and the pancreatic duct, etc. A drainage procedure is indicated in cases of organic papillary narrowing or papillary dysfunction (Figure 6).

*Clinical results*

To date we have performed cholangiomanometry in 744 patients with cholelithiasis and 208 with choledocholithiasis. Figure 7 shows the results in the latter group. A drainage procedure was performed in 8 patients with organic papillary narrowing and 1 with papillary dysfunction, a total of 9 (2.0%). Gallstones recurred in 6 patients. Five of the 6 patients had had abnormal biliary pressures, in whom recurrence could have been prevented if the criteria had been adhered to.

Table 2 summarizes data on biliary drainage procedures performed in our department during the past 28 years. The number of cases during the most 9-year period is smaller than in the preceding periods. This is due to the establishment of well-defined cholangiomanometric criteria. Most importantly, no increase in the incidence of gallstone recurrence or postoperative complaints accompanied this decrease in the number of drainage procedures.

### *Indications for Primary Closure of the Choledochotomy*

In the past, introduction of a T-tube was routine when a choledochotomy was performed. T-tube insertion was thought to be necessary because of papillary dysfunction due to intraoperative manipulation. However, this implies that T-tube

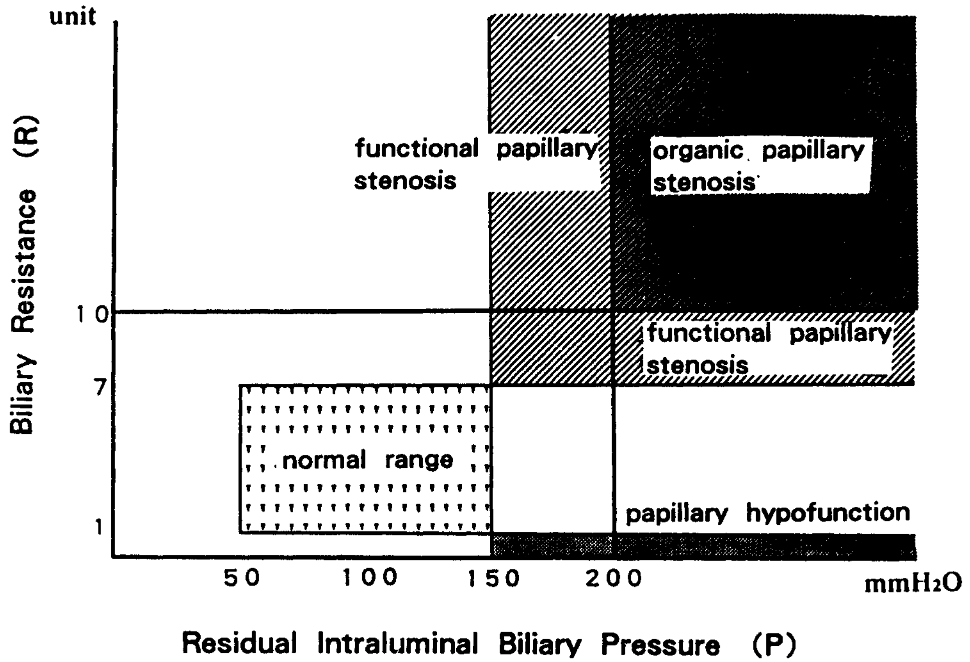

**Figure 6** Classification of papillary dysfunction based on intrabiliary pressure.

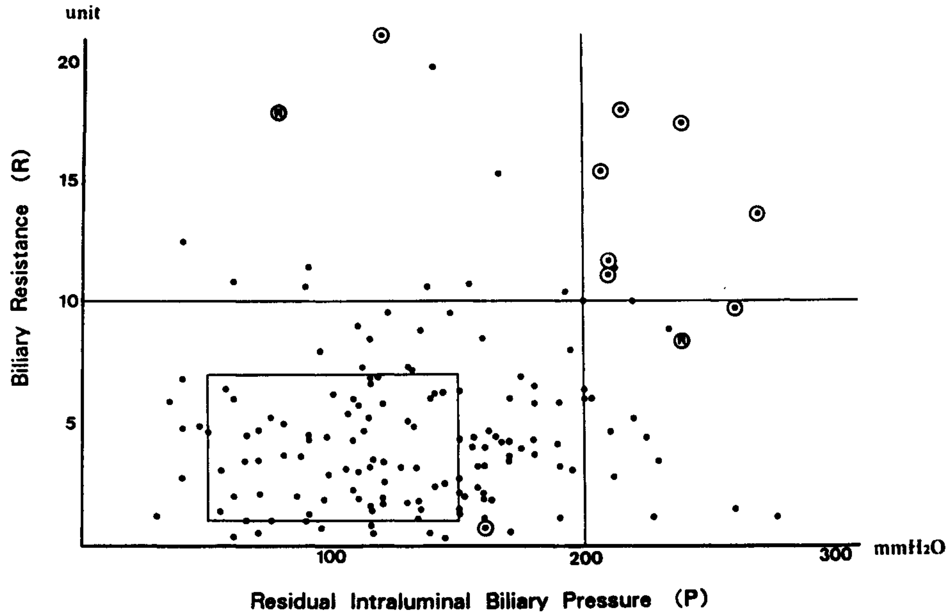

**Figure 7** Intrabiliary pressures in patients with common bile duct stones ($n = 184$). (R), resistant value; (P), residual pressure. O, patients undergoing biliary drainage; R, recurrent stones

**Table 2** The Incidence of biliary drainage procedures in patients with benign biliary disease over time

| | *Number of cases* | *Number of patients undergoing biliary drainage* |
|---|---|---|
| 1960-1972 | 459 | 24 (5.2%) |
| 1973-1978 | 363 | 49 (13.5%) |
| 1979-1991 | 562 | 12 (2.1%) |
| Total | 1384 | 85 (6.1%) |

insertion is not necessary when the common duct and papilla are not disturbed excessively. We employ primary closure without T-tube insertion in patients with normal biliary pressure and 3 or less gallstones on the preoperative cholangiogram. However, in such cases, an intraperitoneal drain must be placed whenever primary closure of the choledochotomy is performed. To date, 21 patients have undergone primary closure successfully. These principles allow even patients with choledocholithiasis to undergo a minilaparotomy and be discharged within one week.

### CONCLUSION

Cholecystectomy associated with minilaparotomy is an established procedure for cholelithiasis which minimizes the extent of surgery in patients with cholecystoand/or choledocholithiasis. The success of this procedure depends on careful preoperative cholangiography and intraoperative examination of the biliary tract. All patients treated by this time limited procedure have experienced a favorable course without complications.

### *References*

1. Danzinger, R.G., Hofmann, A.F. and Schoenfield, L.J. (1972) Dissolution of cholesterol gallstones by chenodeoxycholic acid. *New Eng. J. Med.*, **286**, 1–8
2. Kozu, T., Shimizu, T. and Yamazaki, Y. (1984) Removement of gallstone in biliary tract by YAG laser. *Shokakigeka*, **7**, 587–593 (in Japanese)
3. Sauerbruch, T., Delius, M., and Paumgartner, G. (1986) Fragmentation of gallstones by extracorporeal shock waves. *New Eng. J. Med.*, **314**, 818–822
4. Perissat, J., Collet, D.R. and Belliard, R. (1990) Gallstones: Laparoscopic treatment: cholecystetomy, cholecystostomy and lithotripsy. Our own technique. *Surg.Endosc.*, **4**, 1–5
5. Reddick, E.J. and Olsen, D.O. (1989) Laparoscopic laser cholecystectomy. A comparison with mini-lap cholecystectomy. *Surg. Endosc.*, **3**, 131–139
6. Nagakawa, T., Asano, E., Konishi, I., Higashino, T., Ohta, T., Kanno, M., Akiyama, T. and Miyazaki, I. (1989) Precise operative examinations of the biliary tract: Significance of taking a series of three films for cholangiography during the operation of cholelithiasis and measurement of biliary pressure by variable loading method. *Memoir All. Med. Prof. Kanazawa Univ.*, **13**, 1–7
7. Nagakawa, T., Minai, S., Ueno, K., Ohta, T., Kayahara, M., Akiyama, T., Kadoya, M., Mori, K., Nakano, T. and Miyazaki, I. (1991) Variable loading cholangiomanometry and its clinical application. *Phys. Funct. Bil. Panc.*, **7**, 41–51

*(On invitation by S. Bengmark 22 September 1992)*

